# Cleavage of cellular substrate porcine gasdermin D by porcine torovirus 3C-like protease induces pyroptosis

**DOI:** 10.1080/21505594.2025.2605749

**Published:** 2025-12-16

**Authors:** Dan Pan, Xue-Er Liu, Xin Hong, Yang Liu, Pan-Fu Yin, Jing-Wen Zeng, Qian Lv, En-Zhong Du, Wenchun Fan, Yong-Le Yang, Fushan Shi, Bin Wang, Bo Dong, Yao-Wei Huang

**Affiliations:** a State Key Laboratory for Animal Disease Control and Prevention, South China Agricultural University, Guangzhou, China; b Guangdong Laboratory for Lingnan Modern Agriculture, College of Veterinary Medicine, South China Agricultural University, Guangzhou, China; c Department of Veterinary Medicine, Zhejiang University, Hangzhou, China; d YEBIO Bioengineering Co., Ltd. of Qingdao, Qingdao, China; e Life Sciences Institute, Zhejiang University, Hangzhou, China; f Xianghu Laboratory, Hangzhou, China; g Agro-biological Gene Research Center of Guangdong Academy of Agricultural Sciences, State Key Laboratory of Swine and Poultry Breeding Industry, Guangzhou, China

**Keywords:** Porcine torovirus (PToV), 3C-like protease (3CLP), pyroptosis, gasdermin D (GSDMD), coronavirus

## Abstract

Torovirus (ToV), while resembling coronavirus (CoV), belongs to a distinct family *Tobaniviridae* in the order *Nidovirales*. Porcine ToV (PToV) is widespread in pig populations across many countries, yet its potential pathogenicity in pigs remains poorly understood. The viral 3C-like protease (3CLP) plays a crucial role in processing viral polyproteins and manipulating the host antiviral immune response by targeting cellular proteins through its catalytic activity. In this study, we focused on PToV 3CLP due to its unique catalytic dyad characteristics and substrate recognition properties, which are distinct from those of CoV 3CLPs. We revealed that PToV 3CLP induces pyroptosis in porcine small intestinal IPEC-J2 cells and further demonstrated that porcine gasdermin D (pGSDMD) is a cleavage substrate for PToV 3CLP associated with this process. The catalytic residues, histidine 53 and serine 160, essential for the protease activity of PToV 3CLP, were required for the cleavage of pGSDMD at two distinct sites, glutamine 193 (Q193) and glutamine 277 (Q277). One of fragments produced by PToV 3CLP cleavage, pGSDMD_1–277_, mimicked the activity of the N-terminal domain of pGSDMD (pGSDMD_1–279_) in forming pores and ultimately triggering pyroptosis. Intriguingly, these results contrast with the inhibitory effect of CoV 3CLPs on pyroptosis, previously reported to target pGSDMD at the Q193 site. The study provides additional evidence of the distinct nature of 3CLP between ToV and CoV, which may partly explain the divergent clinical manifestations and pathogenesis observed in pigs infected by these nidoviruses.

## Introduction

The *Nidovirales* order is currently classified into 14 virus families which is recognized by the International Committee on Taxonomy of Viruses (ICTV) [1,2]. The members of two nidovirus families *Coronaviridae* (CoV) and *Arteriviridae* (AV), such as severe acute respiratory syndrome coronavirus (SARS-CoV), SARS-CoV-2, porcine epidemic diarrhea virus (PEDV), porcine deltacoronavirus (PDCoV) and porcine reproductive and respiratory syndrome virus (PRRSV), have been attracting scientific and public attention intensely due to their severe impact on human and animal health [3–8]. The third nidovirus family, *Tobaniviridae*, contains the genus *Torovirus* (ToV) that is also capable of infecting mammals, causing enteritic disorders or subclinical infections in horses, cattle, pigs, goats, and Tibetan antelopes [9–13]. Thus far, equine ToV (EToV), bovine ToV (BToV), and porcine ToV (PToV) have been identified as three distinct species within the subgenus *Renitovirus* of the *Torovirus* genus by ICTV [14]. PToV appears to be widespread in global pig populations, and epidemiological studies have demonstrated high infection rates in swine herds [15–20], with a significant number of these herds also testing positive for co-infections with other swine enteric viruses, such as PEDV [14]. The lack of a PToV cell culture system has impeded our understanding of the PToV biological properties and pathogenesis [14].

ToVs are enveloped, positive-sense, single-stranded RNA viruses with genomes ranging from 25 to 30 kb in size. The ToV genome consists of a core set of genes, including ORF1a, ORF1b, spike (S), membrane (M), hemagglutinin-esterase (HE), and nucleocapsid (N), flanked by 5′- and 3′-untranslated regions [14,21], which share similarities in genome organization, gene order, and replication strategy with CoVs. Viral proteases common among the members of CoV, AV, and ToV, including papain-like proteases (PLP) and 3-chymotrypsin-like proteases (3CLP or 3CLpro), are responsible for processing viral-encoded polyproteins (pp1a from ORF1a and pp1ab from ORF1ab) into different functional non-structural proteins [21–23]. The 3CLP, located in the C-terminal half of pp1a, acts as the main protease (Mpro), carrying out the majority of cleavage events. Although 3CLPs are evolutionally and structurally related, their active-site residues and substrate specificities differ among CoVs, AVs, and ToVs [24]. The AV 3CLP (nonstructural protein 4, nsp4) is a serine protease featuring a typical His-Asp-Ser catalytic triad akin to that of chymotrypsin, and it specifically targets the Glu residue at the P1 position (P1-Glu) for cleavage [25]. However, the 3CLP of CoVs (nsp5) is a cysteine protease characterized by a His-Cys catalytic dyad, with preferences for P1-Gln and P1-Glu, respectively [26,27]. Recently, EToV or PToV 3CLP is determined as a serine protease, resembling that of AVs, yet it utilizes a His-Ser catalytic dyad that specifically recognizes P1-Gln, similar to CoVs [24,28]. Specifically, PToV 3CLP cleaved 12 sites within viral polyproteins at the consensus motif (FXXQ↓A/S; arrow, cleavage site) [28]. Therefore, the ToV 3CLP exhibits combined characteristics from its closest nidoviral relatives.

In addition to processing viral polyprotein, the 3CLP from a range of RNA viruses, including CoVs and AVs, also target cellular proteins through the catalytic activity to subvert host immune response and promote viral infection [29,30]. Viral 3CLPs cleave various critical components and regulatory proteins in the signaling pathways of type I interferon, antiviral stress granules, autophagy, and inflammatory response for degradation [29]. Among these 3CLP-targeted host substrates, key proteins involved in pyroptosis is a notable theme most recently [31–35]. Pyroptosis is a form of programmed cell death that forms pores in the cellular plasma membrane, leading to release of cytoplasmic contents and ultimately curtailing the survival and proliferation of intracellular pathogens [36–38]. Gasdermins (GSDMs), including GSDMA, GSDMB, GSDMC, GSDMD and GSDME play key roles in pyroptosis [36]. As the main executioner of pyroptosis, GSDMD is composed of two domains (N-terminal GSDMD-N, C-terminal GSDMD-C) connected by a peptide linker. Once activated, GSDMD is cleaved at the peptide linker by active caspase-1 in human and caspases 4/5/11 in mice, thus separating GSDMD-N from the auto-inhibitory GSDMD-C, allowing it to bind to membrane lipids and phosphatidylethanolamine to form pores leading to inflammatory programmed lytic cell death [39,40]. Recent studies have shown that 3CLP from CoV, AV, and flavivirus, or 3C protease (3CP) from picornavirus, can cleave GSDMD noncanonically and thereby promote or inhibit pyroptosis, which can be either harmful or beneficial for virus replication [31–35,41].

Thus far, the host cellular substrate and the coupling mechanism that ToV 3CLP regulates have not yet been identified. Given that GSDMD appears to be a conserved and common host target for multiple viral 3CLPs, we aimed to determine whether it is also a substrate for the PToV 3CLP in this study. To this end, we indeed demonstrate that the PToV 3CLP cleave porcine GSDMD (pGSDMD) at two specific sites, glutamine 193 (Q193) and Q277 through its protease activity. The resulting N-terminal fragment, pGSDMD_1–277_, which mimics GSDMD-N, localizes to the cellular membrane and triggers lactate dehydrogenase (LDH) release, bactericidal activity, and pyroptosis. Intriguingly, the finding is in contrast to the inhibitory effect exerted by the CoV 3CLP on this process, providing an additional evidence of the virus-specific trait of 3CLP between ToV and CoV.

## Materials and methods

### Cell culture

Human embryonic kidney HEK-293T cells (ATCC CRL-3216), BSR-T7 cells (BHK-21 cell subclones stably expressing bacteriophage T7 RNA polymerase; kindly provided by Dr Zhenghe Li at Zhejiang University, China), and African green monkey kidney cells (Vero; ATCC CCL-81) were cultured in Dulbecco’s modified Eagle’s medium (DMEM; Gibco), supplemented with 10% fetal bovine serum (FBS; Excell), 100 U/mL penicillin, and 100 μg/mL streptomycin. The porcine small intestinal IPEC-J2 cell line was a generous gift from Dr. Lijuan Yuan at Virginia Tech, Blacksburg, VA. The IPEC-J2 cells were grown in DMEM supplemented with 5% FBS and 1% antibiotics.

### Plasmids, antibodies and inhibitors

The plasmids expressing porcine caspase-1 (with an N-terminal HA-tag), GSDMA, GSDMB, GSDMC, GSDMD, and GSDME (with an N-terminal 3×FLAG-tag) were kindly provided by Dr. Fushan Shi (Zhejiang University, China) [31]. Genes encoding PToV non-structural and structural proteins were amplified from the full-length genomic cDNA clone of the PToV-ZJU39 strain (GenBank accession no. MT684462) [42], and cloned into an expression vector pRK5 with a C-terminal 3×FLAG tag. Site-directed mutagenesis of PToV 3CLP and pGSDMD was carried out using overlap extension PCR. The truncated mutants of pGSDMD were cloned into pCMV vector with 3×FLAG tag at the N-terminus. The DNA fragment containing GFP or PToV 3CLP fused with GFP, linked by a self-cleaving peptide T2A, was digested with SmaI and XhoI and cloned into the pVSV(+)-FL vector, which is an infectious VSV plasmid cDNA (a gift from Dr. Zhenghe Li, Zhejiang University, China). All constructs were confirmed by DNA sequencing.

A polyclonal antibody against PToV 3CLP was generated in-house. Briefly, the *E. coli* codon-optimized sequence of PToV 3CLP, tagged with a 6×His tag at the C-terminus, was cloned and expressed in a pET-28a prokaryotic expression vector. The purified 3CLP protein emulsified with Freund’s complete Adjuvant (Sigma, 344,289) was used to immunize six healthy female 6-week-old BALB/c mice housed in two cages (purchased from Guangzhou Ruige Biological Technology Co., Ltd) via subcutaneous injection (day 0). The animal experiments were approved by the Institutional Animal Care and Use Committee (IACUC) from Guangdong Laboratory Animals Monitoring Institute under the number IACUC2021167, and was carried out from 1 October 2022 to 30 November 2022 (60 days). The mice received two booster immunizations with the 3CLP antigen emulsified with Freund’s incomplete adjuvant (Sigma, 344,291) at 2-week intervals (day 14 and day 28 respectively). Daily checks for injection site reactions, weight loss ( > 15% exclusion) stress behaviors, and food/water intake were performed. Antiserum was harvested and pooled at 30 days post-immunization (day 58) following by measuring the serum IgG titers. Before each procedure, mice were anesthetized with 1.25% isoflurane by inhalation. At the end of the experiment, the mice were euthanized with carbon dioxide. The study adhered to the ARRIVE (Animal Research: Reporting of In Vivo Experiments) guidelines. Anti-FLAG antibody (F1804), and anti-MYC antibody (C3956) were purchased from Sigma. Anti-β-actin antibody was purchased from Abbkine (A01010).

Z-DEVD-FMK (S7312) and disulfiram (S1680) were purchased from Selleck. The optimal concentration of these inhibitors for IPEC-J2 cell viability was determined using a CCK-8 kit (Beyotime Biotechnology, China). IPEC-J2 cells could tolerate the following concentrations of these inhibitors with a 90–100% cell viability level: 10 μM for disulfiram, and 100 μM for Z-DEVD-FMK, respectively. Therefore, we set these values as the maximum concentrations for each inhibitor and established the corresponding concentration gradients.

### Recovery of rVSV expressing GFP or PToV 3CLP

Recombinant VSVs (rVSVs) were recovered by cotransfection of pVSV-GFP or pVSV-3CLP-T2A-GFP, along with auxiliary plasmids encoding VSV nucleocapsid complex (pVSV-N, pVSV-P and pVSV-L) into BSR-T7 cells. At 72 hpt, cells expressing green fluorescence were collected along with the cell culture fluids, and filtered twice through a 0.2-μm filter. Each recombinant virus (rVSV-GFP or rVSV-3CLP-T2A-GFP) was further amplified in Vero cells, and the resultant virus was purified and titrated by plaque assay [43].

### RT-PCR

Viral RNA was extracted from supernatant and lysates of cells infected with rVSV-GFP or rVSV-3CLP-T2A-GFP at 48 hpi using RNAiso Plus reagent (Takara, #9109). The concentration of samples was measured using a NanoDrop spectrophotometer, and reverse transcription was carried out using HiScript III 1^st^ Strand cDNA Synthesis Kit (Vazyme, #R312). The cDNAs were amplified by Phanta-Max Super-Fidelity DNA Polymerase (Vazyme, #P501) and sequenced by GENEWIZ (Guangzhou, China). The primer sequences are available upon request.

### Cell cytotoxicity and cell viability assays

Cell death, indicated by LDH released into the medium, and cell viability were analyzed using a CytoTox 96 nonradioactive cytotoxicity assay kit (Promega, #G1780) and a CellTiter-Glo luminescent cell viability assay (Promega, #G7571), respectively, following the manufacturer’s instructions. For PI staining, HEK-293T or IPEC-J2 cells in 24-well plates were subsequently transfected with plasmids for 24 hours and then washed with cold PBS. The cells were stained with PI (BD Biosciences, #556463) for 15 min at room temperature in the dark and visualized using a fluorescence microscopy (Leica, Germany).

### Immunofluorescence

Cells transfected with plasmids for 48 h or infected with recombinant VSV for 24 h were washed three times with PBS, fixed with 4% paraformaldehyde for 30 min at room temperature, and then washed three times with PBS followed by permeabilization with 0.1% Triton X-100 for 15 min. After washing, the cells were blocked with 5% bovine serum albumin (BSA) for 1 h at 37°C, and then incubated with primary antibody diluted in PBS containing 5% BSA overnight at 4°C. The cells were washed three times with PBS containing 0.05% Tween-20 (PBST) and stained for 1 h with either Alex Fluor 546 goat anti-mouse IgG antibody or Alex Fluor 488 goat anti-rabbit IgG antibody (1:2500; Thermo Fisher Scientific, USA) at 37°C in the dark. Nuclei were stained with 4,’6’-diamidino-2-phenylindole (DAPI; 1:1000; Solarbio, C0060) for 10 min at room temperature, and samples were visualized using a fluorescence microscope or a confocal microscope (Leica, Germany).

### Western blot analysis

Cells were washed twice with cold PBS and then incubated on ice with NP40 cell lysis buffer (Beyotime, P0013F) containing PMSF (Beyotime, ST505). The lysates were denatured for 10 min in 5× loading buffer and separated by sodium dodecyl sulfate-polyacrylamide gel electrophoresis (SDS-PAGE), and the proteins were transferred onto 0.2-μm polyvinylidene difluoride (PVDF) membranes (Millipore, ISEQ00010). After the transfer, the membrane was blocked with 5% *(w/v)* nonfat powdered milk in PBS for 2 h at room temperature and then washed with PBST three times followed by incubation with the primary antibody at 4 °C overnight. After rinsing with PBST, membranes were incubated with the corresponding secondary antibody conjugated with horseradish peroxidase (Fdbio Science, #FD8020) for 1 h at room temperature and detected using an enhanced chemiluminescence kit (NCM Biotech, #P10100). The ladders used in the western blot were purchased from Vazyme (cat#MP201).

### Co-immunoprecipitation assay

HEK-293T cells seeded in 6-well plates were transfected with the specific plasmids. At 24 h after transfection, cells were lysed with NP40 cell lysis buffer (Beyotime, P0013F) containing PMSF (Beyotime, ST505). The lysates were centrifuged at 4°C for 15 min, and the supernatants were incubated with anti-FLAG-binding Dynabeads Protein G (Thermo Fisher Scientific, 10004D) at 4°C overnight. The Dynabeads were then washed 5 times with PBST and denatured in 1× SDS-PAGE loading buffer for 10 min. Finally, the resulting samples were analyzed by immunoblotting.

### Expression of truncated pGSDMD fragments in E. coli

Full length pGSDMD (WT) and truncated pGSDMD variants (pGSDMD_1–279_, pGSDMD_1–193_, pGSDMD_1–277_, pGSDMD_194–277_, pGSDMD_194–488_, and pGSDMD_278–488_) were cloned into pCold-TF prokaryotic expression vector, respectively. *E. coli* BL21 cells transformed with these plasmids were grown in Luria-Bertani (LB) medium supplemented with 50 μg/mL kanamycin. Protein expression was induced with 0.25 mM IPTG at 16°C overnight after optical density at 600 nm (OD_600_) reached 0.8. The cells were harvested and lysed by sonication on ice in PBS containing PMSF, followed by centrifugation at 12,000 *g* at 4°C for 30 min. The supernatant was collected and then the expressed products were analyzed by SDS-PAGE with Coomassie blue staining.

### Sequence alignment

We collected aa sequences of pp1a containing 3CLPs from PToV (GenBank accession no. QUU43912.1), BToV (GenBank accession no. YP_337905.2), EToV (GenBank accession no. YP_009665195.1), and CToV (GenBank accession no. YP_009380536.1), and those of pGSDMD (GenBank accession no. XP_020946165.1) and other GSDMD homologues from bovine (GenBank accession no. NP_001346905.1), equine (GenBank accession no. XP_023504873.1), caprine (GenBank accession no. ALN66870), and human (GenBank accession no. NP_001159709.1). SnapGene software was used to perform the multiple-sequence alignment.

### Statistical analysis

The experiments involved in the whole study was performed from September 2022 to August 2025. All experiments were conducted in triplicate. Data are represented as mean ± standard deviation using GraphPad Prism 9.3. Differences between groups were evaluated with Student’s *t*-test using SPSS software for Windows (version 20.0; SPSS, Inc., Chicago, IL, USA). Differences were considered statistically significant at *p* ≤ .05.

## Results

### Expression of specific PToV proteins, including 3CLP, induces pyroptosis associated with pGSDMD cleavage in porcine IPEC-J2 cells

**Figure 1.**
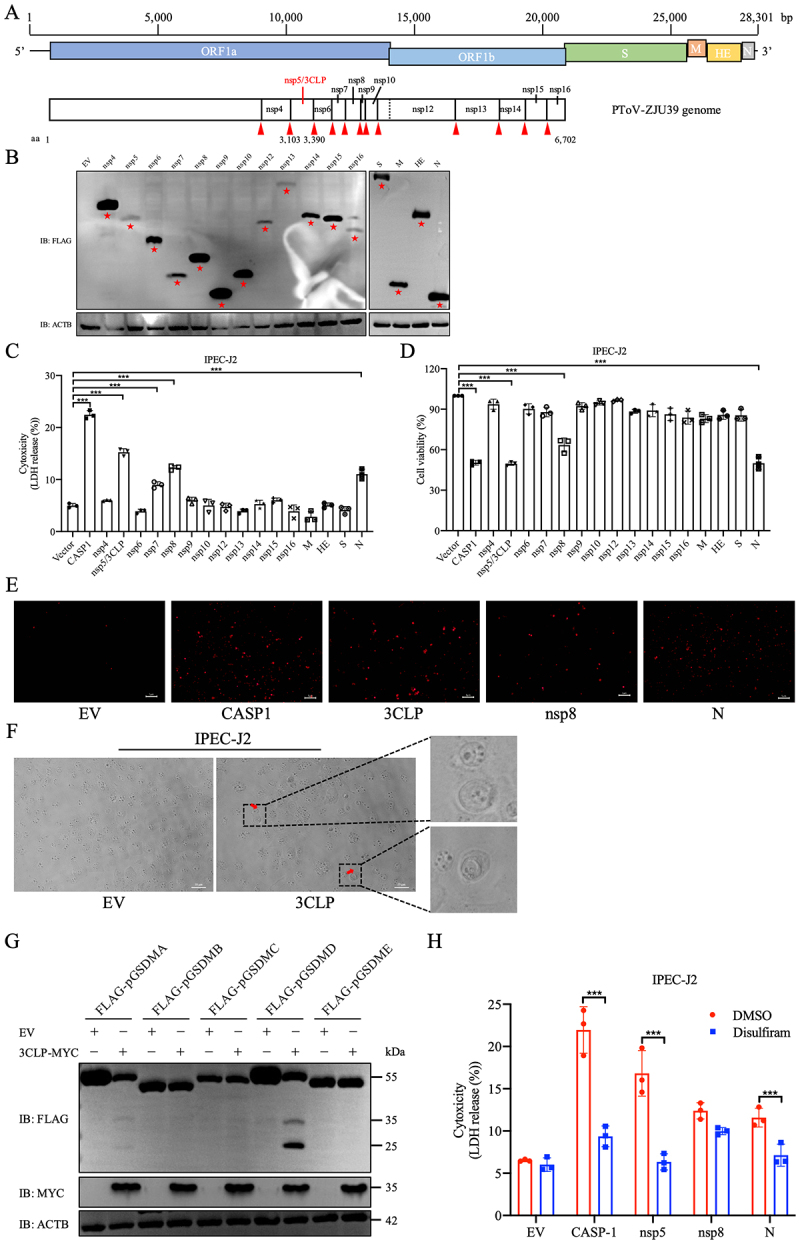
Screening of viral proteins encoded by PToV that can induce pyroptosis associated with pGSDMD *in vitro*. (A) Schematic diagram of genomic structure of the PToV-ZJU39 strain (GenBank accession no. MT684462) and the putative domains cleaved by 3CLP (nsp5) within in ORF1a and ORF1ab corresponding to the nomenclature of sixteen CoV nonstructural proteins. The numbers on the scale bar indicate distances from the 5’ end of the genome. The 3CLP cleavage sites are also marked by red arrowheads. (B-D) Porcine IPEC-J2 cells were transfected with constructs expressing a total of 16 viral proteins (twelve nonstructural proteins: nsp4 to nsp10, and nsp12 to nsp16; and four structural proteins: S, he, M and N) from the PToV-ZJU39 strain. Empty vector (EV) and plasmid carrying porcine caspase-1 (CASP1) were used as negative and positive controls, respectively. Detection of each of sixteen PToV proteins (indicated by red stars) was conducted in IPEC-J2 cells at 36 h post-transfection (hpt) by western blot B). At 36 hpt, the supernatants were collected and analyzed for LDH release (C) and ATP-based cell viability (D). LDH release and cell viability are expressed as means ± SD from three technical replicates. All the shown data are representative of three independent experiments. ***p < 0.001. (E and F) Pyroptotic cell death was detected by PI staining (E; Scale bars, 5 μm), and was observed by morphological changes (F; Scale bars, 10 μm), in IPEC-J2 cells transfected with expression plasmids encoding CASP1, PToV-nsp5, -nsp8, or -N protein. Typical pyroptotic features are indicated by red arrows and highlighted in zoomed regions in cells expressing 3CLP (F). (G) Detection of potential proteolysis of five GSDMD members upon the addition of 3CLP. HEK-293T cells were co-transfected with a plasmid expressing FLAG-tagged pGSDMA, pGSDMB, pGSDMC, pGSDMD or pGSDME, along with a plasmid expressing PToV 3CLP with a myc tag or EV. Western blot analysis was performed at 24 hpt. (H) After IPEC-J2 cell treated with disulfiram (final concentration of 0.5 μM) or DMSO for 2 h, EV or plasmids expressing CASP1, PToV nsp5, nsp8, or N were transfected into IPEC-J2 cell. The supernatants were collected and analyzed for LDH levels at 36 hpt. ***p < 0.001.

Based on the previously identified 3CLP cleavage sites within the ORF1a and ORF1ab [28], and the four known structural protein genes (S, HE, M and N) of PToV [42], we first constructed a set of expression plasmids carrying specific PToV viral genes that encode 12 nonstructural proteins (nsp4 to nsp10, and nsp12 to nsp16, corresponding to the nomenclature of sixteen CoV nsp), as well as S, HE, M, and N (Figure 1(A)). Notably, nsp5 is equivalent to ToV 3CLP according to its counterpart named in CoV. To screen which viral proteins induce pyroptotic cell death, these constructs, along with an empty vector (EV) and an expression construct of porcine caspase-1 (CASP1), which is known to play a critical role in pyroptosis induction through the cleavage of GSDMD [31,36], were each transfected into the porcine small intestinal IPEC-J2 cells. This cell line has been used for the study of type I interferon signaling and inflammatory response in porcine virus infection previously [44]. Expression of each of 16 proteins was verified by western blot analysis (Figure 1(B)). Subsequently, the releasing amount of LDH was evaluated at 36 hours post-transfection (hpt), as the presence of extracellular LDH from the cytosol is widely recognized as an indicator of pyroptosis [36–38]. We observed that the LDH release from IPEC-J2 cells expressing CASP1, nsp5 (3CLP), nsp7, nsp8, and N increased significantly, with nsp5 inducing a greater LDH release than the other three PToV proteins (Figure 1(C)). Accordingly, cell viability markedly decreased after transfection with plasmids expressing CASP1, nsp5 (3CLP), nsp8, or N, except for nsp7 (Figure 1(D)). To further confirm the nature of cell death in CASP1-, 3CLP-, nsp8- and N-expressing cells, we performed a membrane-impermeant dye, propidium iodide (PI), staining assay. Consistently, overexpression of PToV 3CLP, nsp8 and N, as well as CASP1 increased the percentage of PI-positive staining cells that loss cell membrane integrity (Figure 1(E)). In addition, the morphological changes in cells expressing 3CLP displayed typical pyroptotic features, including cell swelling, rounding, and “fried-egg” appearance due to the protruding nucleus (Figure 1(F)), which were distinguished from apoptotic cells showing nuclear condensation, cytoplasmic vaculation, and plasma membrane blebbing, and necroptotic cells showing lytic morphology with bursting extensions [40]. The screening results indicated that expression of PToV nsp5 (3CLP), nsp8 or N, but not other viral proteins, induces pyroptosis in porcine IPEC-J2 cells.

Since pyroptosis is commonly induced by the GSDM family, to further determine which GSDMs are involved in the regulation of pyroptosis by PToV 3CLP, we co-expressed each GSDM member with a FLAG tag and 3CLP with a MYC tag (3CLP-MYC) or EV in transfected cells and examined their proteolysis. At 24 hpt, in the presence of 3CLP-MYC, full-length pGSDMD (55 kDa), but not pGSDMA, pGSDMB, pGSDMC, or pGSDME, was cleaved into two distinct bands with molecular masses of approximately 25 and 35 kDa, respectively, accompanied by a reduced abundance (Figure 1(G)). We also noticed a decrease in GSDMA levels upon the addition of 3CLP-MYC, and further investigation is warranted to elucidate the underlying mechanism. To determine whether the cleavage of pGSDMD by 3CLP could induce or inhibit cell pyroptosis, IPEC-J2 cells were pretreated with dimethyl sulfoxide (DMSO; as a mock control) or disulfiram, which is known to specifically inhibit the pore-forming activity of GSDMD [45]. They were then transfected with nsp5 (3CLP) or other controls, including EV, CASP1, nsp8, and N. Compared to the DMSO-treated group, the 3CLP-expressing cells treated with disulfiram showed a significantly lower LDH level amounted to the EV control at 36 hpt (Figure 1H), suggesting that inactivation of pGSDMD inhibit 3CLP-induced pyroptosis. These data indicate that PToV 3CLP likely trigger the activation of GSDMD by its cleavage, which leads to pyroptosis. The mechanism by which PToV nsp8 and N induce pyroptosis remains to be further investigated.

### A recombinant VSV expressing PToV 3CLP induces pyroptosis in IPEC-J2 cells

**Figure 2.**
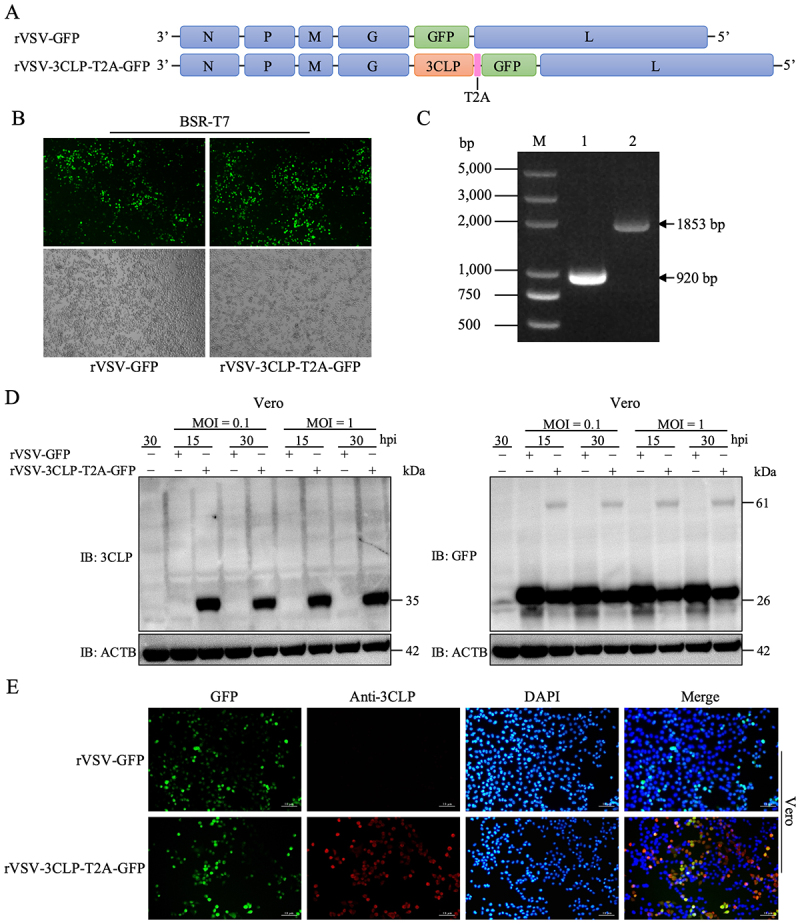
Rescue and characterization of recombinant vesicular stomatitis virus (rVSV) expressing PToV 3CLP. (A) Schematic diagram of recombinant VSV expressing GFP (rVSV-GFP) or 3CLP fused with GFP (rVSV-3CLP-T2A-GFP). (B) rescue of two rVsvs from full-length rVSV clone plasmid plus the helper plasmids expressing VSV-N, -P and -L in BSR-T7 cells. GFP signal was monitored by fluorescence microscopy at 72 hpt in BSR-T7 cells. (C) rVSV expression of 3CLP was confirmed by RT-PCR. RNA was extracted from infected Vero cells, and RT-PCR was used to amplify the region spanning between the G and L gene. Lane m, marker; lane 1, rVSV-GFP; lane 2, rVSV-3CLP-T2A-GFP. (D) Analysis of VSV expression of 3CLP using a specific anti-3CLP (left panel) or an anti-GFP antibody (right panel). Vero cells in 6-well were infected with either rVSV-GFP or rVSV-3CLP-T2A-GFP at MOI of 0.1 or 1.0. At the indicated times, cells were lysed and analyzed by western blot. (e) IFA detection of GFP or PToV 3CLP proteins in Vero cells using anti-3CLP polyclonal antibody at 24 hpi. Scale bars, 10 μm.

Since PToV has not yet been isolated in cultured cells, a recombinant vesicular stomatitis virus (rVSV) system was employed as a surrogate model to verify 3CLP-induced pyroptosis in the infection context. We first inserted the coding sequence of green fluorescent protein (GFP) as a control, or PToV 3CLP fused with GFP via a self-cleaving T2A peptide linker (3CLP-T2A-GFP), into the VSV backbone between glycoprotein (G) gene and the large (L) polymerase gene (Figure 2(A)). We then recovered two corresponding recombinant viruses, rVSV-GFP and rVSV-3CLP-T2A-GFP in BSR-T7 cells by reverse genetics as described previously [46]. Green fluorescence became clearly observable at 72 hpt (Figure 2(B)), and the two recombinant rVSVs could be passaged serially in Vero cells. RT-PCR of the region between the VSV G and L genes confirmed the correct insertion of fragments of 920 bp and 1860 bp for rVSV-GFP and rVSV-3CLP-T2A-GFP, respectively (Figure 2(C)). To further examine the expression of 3CLP and GFP proteins, Vero cells were inoculated with each rVSV at a multiplicity of infection (MOI) of 0.1 or 1.0, and cell lysates were harvested at 15 or 30 h post-infection (hpi). Proteins were detected by western blot using a polyclonal antibody against PToV 3CLP (anti-3CLP) that was generated in-house. A 35-kDa protein band corresponding to the size of 3CLP was detected exclusively in cells infected with rVSV-3CLP-T2A-GFP, but not in the rVSV-GFP-infected control cells (Figure 2(D), left panel). Utilizing an anti-GFP antibody revealed protein bands of 61 kDa and 26 kDa in rVSV-3CLP-T2A-GFP-infected cells, representing the noncleaved 3CLP fused with GFP and GFP self-cleaved by T2A, respectively (Figure 2D, right panel). Meanwhile, Vero cells infected with each rVSV were examined by immunofluorescence assay (IFA) using anti-3CLP antibodies. The results showed colocalization of GFP and anti-3CLP staining specifically in rVSV-3CLP-T2A-GFP-infected cells, thereby further confirming expression of 3CLP by this recombinant virus (Figure 2(E)).

**Figure 3.**
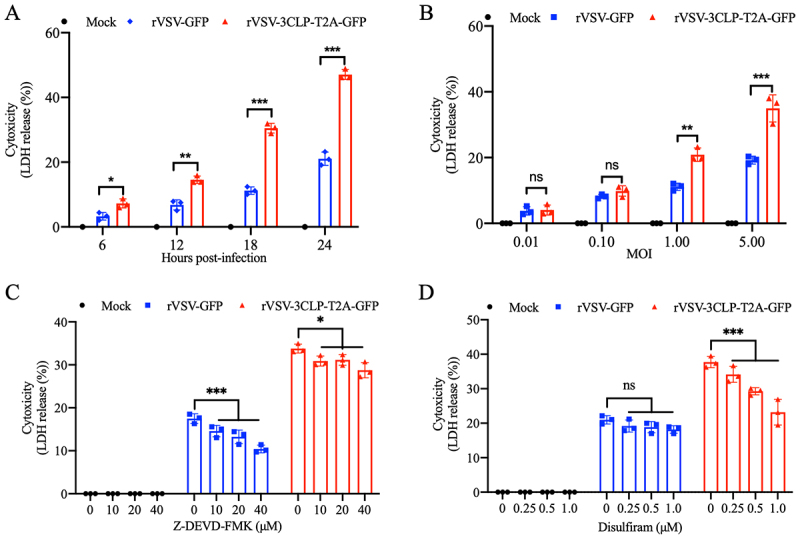
Recombinant VSV expressing PToV 3CLP induces pyroptosis in IPEC-J2 cells. (A) IPEC-J2 cells were mock infected or infected with each rVSV at a MOI of 5. At the indicated times, the supernatants were collected and analyzed for LDH level. (B) IPEC-J2 cells were mock infected or infected with each rVSV at MOI of 0.01, 0.1, 1, or 5. At 24 hpi, the supernatants were collected and analyzed for LDH level. (C). IPEC-J2 cells were mock infected or infected with each rVSV at MOI of 5 along with Z-DEVD-FMK treatment (final concentrations 0 μM, 10 μM, 20 μM and 40 μM). At 24 hpi, the supernatants were collected and analyzed for LDH level. (D) IPEC-J2 cells pretreated with disulfiram (final concentration 0 μM, 0.25 μM, 0.5 μM and 1.0 μM) were mock infected or infected with each rVSV at MOI of 5. At 24 hpi, the supernatants were collected and analyzed for LDH level. All the shown data are representative of three independent experiments. *p < 0.05; ***p < 0.001; ns: not significant.

To determine whether PToV 3CLP in an infectious rVSV context induces greater pyroptosis than the control without 3CLP (as VSV infection also triggers cell pyroptosis; see the next paragraph), time course experiments were carried out in IPEC-J2 cells infected with rVSV-GFP or rVSV-3CLP-T2A-GFP at a MOI of 5. While both rVSV infections potentiated the release of LDH, those infected with rVSV-3CLP-T2A-GFP had significantly greater LDH release compared with the rVSV-GFP control, and LDH release continuously increased with time (Figure 3(A)). Consistently, cells infected with rVSV-3CLP-T2A-GFP at two MOI values (1 and 5) had significantly higher LDH release at 24 hpi compared with cells infected with the rVSV-GFP control (Figure 3(B)).

VSV infection is known to activate caspase-3, which then cleaves GSDME and induces cell pyroptosis [47]. To offset the effect of caspase-3 on the observed cytotoxicity, the caspase-3 inhibitor, Z-DEVE-FMK, was used to treat infected cells at different concentrations. Z-DEVD-FMK treatment exhibited a significant dose-dependent reduction in LDH release in IPEC-J2 cells infected by both rVSVs at 24 hpi (Figure 3(C)). However, rVSV-3CLP-T2A-GFP still induced a higher level of pyroptosis than the rVSV-GFP control, suggesting that this effect was attributable to 3CLP. Finally, a specific GSDMD inhibitor, disulfiram, was used to treat IPEC-J2 cells infected with the rVSVs. Disulfiram treatment of rVSV-3CLP-T2A-GFP-infected cells reduced LDH release in a dose-dependent manner at 24 hpi, whereas cells infected with rVSV-GFP did not change significantly (Figure 3(D)). Taking together, the results demonstrate that PToV 3CLP, driven by rVSV infection, induces a potent and specific GSDMD-mediated pyroptosis that is independent of the VSV-triggered and GSDME-mediated cell pyroptosis in IPEC-J2 cells.

### The protease activity of PToV 3CLP is required for the cleavage of pGSDMD

**Figure 4.**
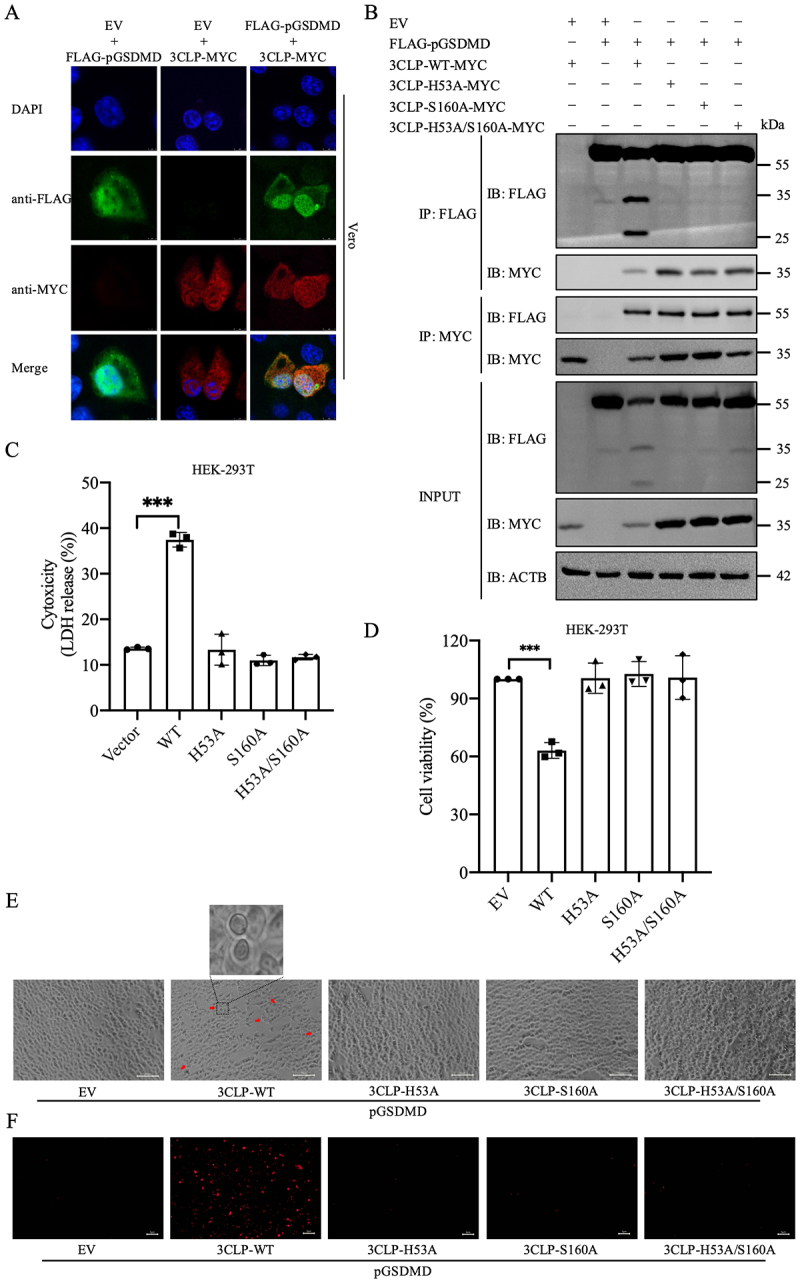
The protease activity of PToV 3CLP is crucial for cleavage of pGSDMD to induce pyroptosis. (A) Vero cells were co-transfected with plasmids expressing PToV 3CLP-MYC and FLAG-tagged pGSDMD, or with an empty vector (EV) and FLAG-tagged pGSDMD (or 3CLP-MYC) for 24 h. The cells were probed with anti-FLAG (green) and anti-MYC (red). The cell nuclei (blue) were stained with DAPI. Scale bars, 5 μm. (B-F) HEK-293T cells were co-transfected with plasmids expressing FLAG-tagged pGSDMD and either wild-type (WT) PToV 3CLP-MYC or its protease-defective mutants (H53A, S160A and H53A/S160A). At 24 hpt, cells were lysed for co-IP detection (B); cell supernatants were collected and analyzed for LDH levels (C); cell viability was evaluated (D); cell morphology was observed using a Leica microscope (e; scale bars, 10 μm); and cells were stained with PI (4F; scale bars, 5 μm). Typical pyroptotic features are indicated by red arrows and highlighted in zoomed regions in cells expressing 3CLP-WT (E). All the data are representative of three independent experiments. ***p < 0.001.

To confirm if PToV 3CLP indeed interacts with pGSDMD, we co-transfected Vero cells with 3CLP-MYC and FLAG-pGSDMD. At 24 hpt, we observed colocalization of 3CLP-MYC with pGSDMD in both the cytoplasm and the cell nucleus (Figure 4(A)). It has been reported that PToV 3CLP employs His-53 and Ser-160 as the proteolytic-active sites [28]. Thus, to explore whether PToV 3CLP cleaves pGSDMD by means of its protease activity, we constructed three plasmids expressing different mutants of PToV 3CLP: H53A, S160A and H53A/S160A, and used them to assess pGSDMD cleavage (Figure 4(B)). Western blot analysis clearly revealed the same cleavage pattern of pGSDMD as observed in Figure 1(G), exclusively in cells co-transfected with wild-type (WT) PToV 3CLP, but not in those transfected with 3CLP mutants (Figure 4(B)). In addition, co-immunoprecipitation (co-IP) experiments demonstrated that both wild-type and mutant PToV 3CLPs interacted with pGSDMD, suggesting that these mutants lost the ability to cleave pGSDMD but retained their ability to bind pGSDMD (Figure 4(B)). Furthermore, significantly increased LDH release and reduced cell viability was only detected in cells co-transfected with PToV 3CLP-WT and pGSDMD, and not after co-transfection with the 3CLP mutants (Figure 4(C,D)). Cells co- transfected with PToV 3CLP-WT and pGSDMD exhibited typical pyroptotic features at 24 hpt, whereas none of the three mutant PToV 3CLPs changed cell morphology (Figure 4(E)). Consistently, the percentage of positive PI-staining cells increased in HEK-293T cells co-transfected with 3CLP-WT and pGSDMD, but not those transfected with the mutant 3CLPs (Figure 4(F)). Therefore, substitutions in the active site of PToV 3CLP disrupted its protease activity, resulting in the failure to cleave pGSDMD. Consequently, the protease activity of PToV 3CLP is essential for the cleavage of pGSDMD and the subsequent induction of pyroptosis.

### PToV 3CLP cleaves pGSDMD at residues Q193 and Q277

**Figure 5.**
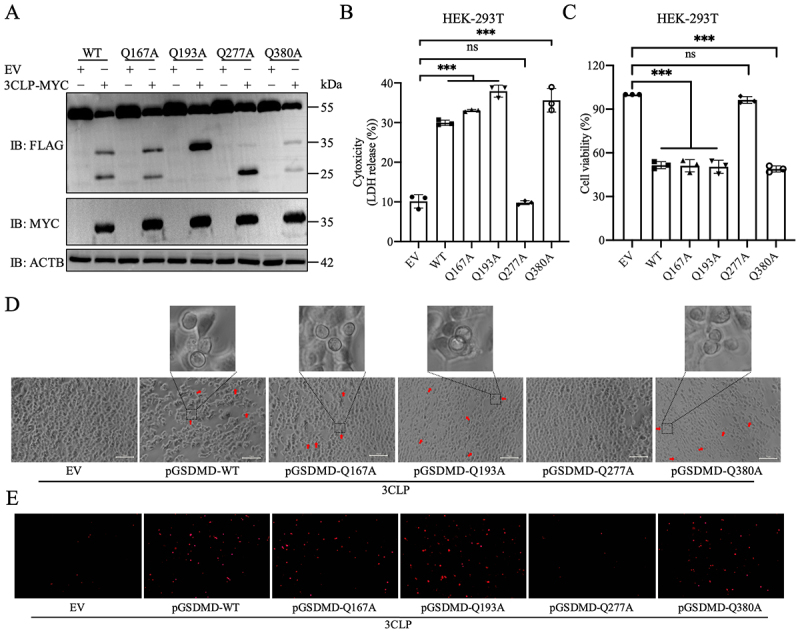
pGSDMD is cleaved at residues Q193 and Q277 by PToV 3CLP. HEK-293T cells were co-transfected with plasmids expressing PToV 3CLP-MYC and FLAG-tagged pGSDMD-wt or its various mutants (Q167A, Q193A, Q277A and Q380A). At 24 hpt, cells were lysed for western blot (A); cell supernatants were collected and analyzed for LDH levels (B); cell viability was evaluated (C); cell morphology was observed using a Leica microscope (D; Scale bars, 10 μm); and cells were stained with PI (e; scale bars, 5 μm). Typical pyroptotic features are indicated by red arrows and highlighted in zoomed regions (D). All the data are representative of three independent experiments. ***: *p* < 0.001.

According to a previous report that PToV 3CLP cleaves the viral polyproteins at the consensus sequences (FXXQ↓A/S) ^28^, we predicted the potential cleavage sites within pGSDMD (GenBank accession no. XM_021090506). Based on the sizes of the cleaved bands of approximately 25- and 35-kDa (Figure 1(F) and 4(B)), we deduced the following sites within pGSDMD for further testing as the potential proteolytic sites for PToV 3CLP: Q167-K168, Q193-G194, Q277-S278, and Q380-A381. We then constructed four single-substitution pGSDMD mutants in which glutamine was replaced by alanine, namely Q167A, Q193A, Q277A, and Q380A, to assess pGSDMD cleavage. pGSDMD-WT or its mutants were co-expressed with 3CLP-MYC or EV in HEK-293T cells. Cleavage of pGSDMD-WT, -Q167A, and -Q380A by PToV 3CLP resulted in two bands of approximately 25- and 35-kDa, both representing the FLAG-tagged N-terminal fragment of pGSDMD (Figure 5(A)). However, co-expression of either the Q193A or Q277A mutant with PToV 3CLP led to the disappearance of a single band: the 25-kDa band for Q193A and the 35-kDa band for Q277A, indicating a loss of a cleavage site (Figure 5(A)).

The effects of these pGSDMD mutants on the induction of pyroptosis were further assessed. LDH release was augmented, and cell viability was reduced in HEK-293T cells co-transfected with 3CLP and pGSDMD-WT, -Q167A, -Q193A or -Q380A, but not with pGSDMD-Q277A (Figure 5(B,C)). Consistently, morphological changes and the percentage of PI-positive staining in co-transfected cells varied depending on the pGSDMD variant used. Cells co-transfected with pGSDMD-WT, -Q167A, -Q193A or -Q380A exhibited typical pyroptotic appearance (Figure 5(D)) and higher numbers of PI positivity (Figure 5(E)), whereas those co-transfected with pGSDMD-Q277A did not show altered morphology or PI staining. These results collectively demonstrate that Q193 and Q277 within pGSDMD are the cleavage sites of PToV 3CLP, and that failure to cleave pGSDMD at Q277 prevents the induction of pyroptosis.

### pGSDMD_1–277_ induces pyroptosis and exhibits bactericidal activity

**Figure 6.**
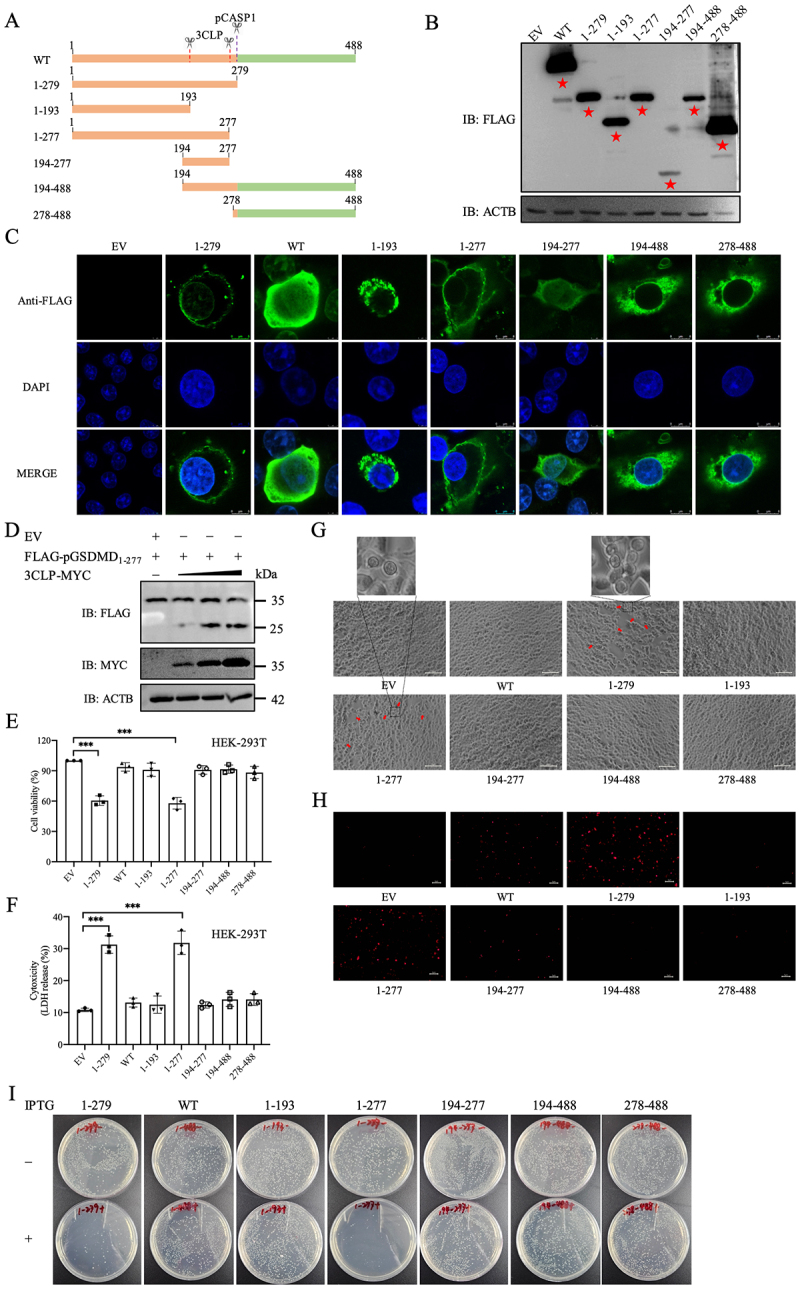
Characterization of pGSDMD cleavage fragments in pyroptosis induction and bactericidal activity. (A) Schematic diagram illustrating the N-terminal and C-terminal domains of pGSDMD and the cleavage fragments generated by PToV 3CLP (at Q193 and Q277) or pCASP1 (at D279). (B) Detection of the expression of the pGSDMD constructs (indicated by red stars) in transfected cells by western blot. (C) Vero cells were transfected with plasmids encoding FLAG-tagged pGSDMD or its cleavage fragments: pGSDMD_1–279_, pGSDMD_1–193_, pGSDMD_1–277_, pGSDMD_194–277_, pGSDMD_194–488_ and pGSDMD_278–488_. At 24 hpt, cells were fixed and subsequently immunostained with an anti-FLAG antibody, followed by staining with Alexa Fluor-488 antibody (green) and nuclear staining with DAPI (blue). Immunofluorescence signals were observed using a confocal microscope. Scale bars, 8 μm. (D) HEK-293T cells were cotransfected with plasmids encoding FlAG-pGSDMD_1–277_ and various doses of 3CLP-MYC. After 24 h, cells were lysed for immunoblotting assay. (E to H) HEK-293T cells were transfected with plasmids encoding FLAG-tagged pGSDMD or its cleavage fragments. At 24 hpt, cell supernatants were collected and analyzed for LDH levels (E); Cell viability was evaluated (F); cell morphology was observed using a Leica microscope (G; scale bars, 10 μm); and cells were stained with PI (H; Scale bars, 5 μm). Typical pyroptotic features are indicated by red arrows and highlighted in zoomed regions (G). Data represent of three independent experiments. ***p < 0.001. (I) *E. coli* BL21 was transformed with pCold-TF harboring the indicated pGSDMD cleavage fragments. Cells were diluted and grown on LB agar plates containing 50 μg/ml kanamycin with or without 0.25 mM IPTG. After incubation overnight, bacterial colonies on the plates were visualized and evaluated.

Previous studies have shown that porcine CASP1 cleaves pGSDMD at D279, producing pGSDMD_1–279_, which localizes to the cellular membrane and induces pyroptosis by forming pores [31,33]. Based on this, we hypothesized that cleavage of pGSDMD at Q277 May mimic the behavior of pGSDMD_1–279_ (Figure 6(A)). To further investigate the role of pGSDMD fragments cleaved by PToV 3CLP in pyroptosis induction, we constructed five FLAG-tagged truncated pGSDMD mutants: pGSDMD_1–193_, pGSDMD_1–277_, pGSDMD_194–277_, pGSDMD_194–488_, and pGSDMD_278–488_, which were equivalent in length to the cleavage fragments generated by PToV 3CLP (Figure 6(A)). The expression and molecular size of these truncated proteins were confirmed by western blot in transfected cells (Figure 6(B)). We then visualized the cellular distribution of expressed full-length pGSDMD-WT and the truncated pGSDMD mutants in Vero cells. The pGSDMD_1–277_ fragment primarily localized to the cellular membrane (Figure 6(C)), similar to the subcellular localization of pGSDMD_1–279_ as previously reported [31,33], while the other truncated pGSDMD fragments were diffusely distributed in the nucleus and cytosol (Figure 6(C)). To determine whether PToV 3CLP could continue to cleave pGSDMD_1–277_ at Q193, HEK-293T cells were co-transfected with 3CLP and pGSDMD_1–277_. As shown in Figure 6(D), cleavage of pGSDMD_1–277_ progressively increased in a PToV 3CLP dose-dependent manner. HEK-293T cells transfected with the plasmid encoding pGSDMD_1–277_, as well as the positive control pGSDMD_1–279_, promoted LDH release (Figure 6(E)) and decreased cell viability (Figure 6(F)). Accordingly, we found that pGSDMD_1–277_, resembling pGSDMD_1–279_, exhibited typical pyroptotic morphological changes (Figure 6(G)). PI staining showed a higher number of PI-positive HEK-293T cells transfected with plasmids encoding pGSDMD_1–277_ and pGSDMD_1–279_ (Figure 6(H)).

A previous study has also shown that human GSDMD_1–275_ (GSDMD-N; equivalent to pGSDMD_1–279_) exhibits bactericidal activity through its membrane pore-forming feature [48]. To assess the bactericidal activity of pGSDMD fragments cleaved by PToV 3CLP, we cloned the truncated regions of pGSDMD into the prokaryotic vector pCold-TF and expressed them in the *Escherichia coli* (*E. coli*) BL21 strain by isopropyl β-D-1-thiogalactopyranoside (IPTG) induction. Upon induction with IPTG, *E. coli* counts significantly reduced with the expression of pGSDMD_1–279_ and pGSDMD_1–277_, but not with the expression of pGSDMD-WT or other PToV 3CLP-mediated cleaved pGSDMD fragments (Figure 6(I)). The finding suggests that pGSDMD_1–277_, similar to pGSDMD_1–279_, likely forms membrane pores that are detrimental to bacteria. Taken together, these results confirm our hypothesis that, since Q277 is close to the CASP-1 cleavage site D279 within pGSDMD, the cleavage product pGSDMD_1–277_ by PToV 3CLP resembles pGSDMD_1–279_ in inducing subsequent cell pyroptosis.

### Analysis of interaction between ToV 3CLP and GSDMD within related viral and host species

**Figure 7.**
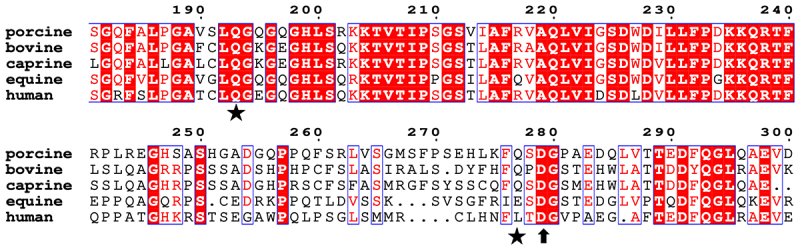
Amino acid (aa) sequence alignment of the cleaved regions in GSDMDs from five different animal species: porcine, bovine, caprine, equine, and human. The two PToV 3CLP cleavage sites at aa positions 193 and 277 are indicated by asterisks, and the porcine caspase-1 cleavage site at aa position 279 is marked by a triangle.

To better understand the potential ability of distinct 3CLPs from various ToV species to cleave GSDMDs from their corresponding host species, we analyzed the amino acid sequences (aa) of four known ToV (PToV, BToV, EToV, and caprine ToV – CToV) 3CLPs and five GSDMDs (from porcine, bovine, equine, and caprine hosts, and human). The PToV 3CLP (ZJU39 stain) is 287 aa in length, which shares 86.76%, 77.35%, and 86.41% aa sequence identities with the 3CLPs from BToV (287 aa), EToV (292 aa), and CToV (287 aa), respectively. Moreover, a significant homology was identified among different reservoir-original ToV 3CLPs, including the conserved active sites (His-53 and Ser-160 catalytic dyad), suggesting that BToV, EToV, and CToV 3CLPs likely recognize the same host homologous substrates as PToV 3CLP. Though a multiple sequence alignment revealed significant similarity among the GSDMDs from five different species, including the first PToV 3CLP cleavage site at Q193 and the caspase-1 cleavage site at D279, only bovine and caprine GSDMDs harbor the second putative 3CLP cleavage site at Q277, as seen in pGSDMD (Figure 7). It is likely that both could be cleaved at Q277 by BToV and CToV 3CLPs. Given the distinct amino acid residues at this key cleavage site in equine and human GSDMDs (E and L), we speculate that these GSDMDs are unlikely to be cleaved in this position by the four known ToV 3CLPs.

## Discussion

Although ToV was previously classified within the CoV family and remains most closely related to CoVs in the order *Nidovirales*, the molecular biology of ToV has been poorly studied [14,21]. Thus far, only a few ToV species and their corresponding full-length genomes have been identified, including EToV, BToV, PToV, CToV, and antelope ToV [13,24,28,42,49]. Most of our current knowledge about this family primarily comes from research on EToV and BToV, both of which can be grown in cell culture [21]. Recently, PToV has received increasing attention, driven by the findings that inter-order recombination events between PToV and porcine enterovirus G (EV-G) have occurred worldwide [14]. Most recently, our group developed a novel serological detection ELISA based on the PToV S1 protein and revealed a relatively high incidence of seropositivity in diarrheic pigs of different ages, providing the first serological evidence for PToV infection in pigs from eastern China [42]. We also identified and sequenced a complete PToV genome from fecal samples collected from one PToV-seropositive swine herd in Zhejiang province, China [42]. To elucidate the potential pathogenicity of PToV in pigs, we set up to investigate the roles of critical PToV proteins that are homologous to their CoV counterparts in virus-host interaction. In this study, we focused on PToV 3CLP because 3CLP not only cleaves viral replicase polyproteins to produce viral nsps involved in the viral life cycle [28], but may also cleave cellular substrates to modulate host immune responses [29]. Moreover, the ToV 3CLP possesses unique catalytic dyad characteristics and substrate recognition properties that distinguish it from CoV and AV 3CLPs [24,28]. To this end, we have identified the first cellular substrate, pGSDMD, of 3CLP from PToV, which has not been previously reported among members of the ToV family, and further demonstrated the consequence of cleaving pGSDMD to induce cell pyroptosis.

Interestingly, another viral protease of ToV, PLP, has been identified to partially insert into the 2C/3A junction of the porcine EV-G genome, likely functioning as a deubiquitinase and deISGylase to suppress host cell innate immune responses [50]. This could potentially allow recombinant EV-G to improve its ability to evade host immunity. However, the specific ubiquitin-conjugated and ISGylated protein substrates for this ToV-like PLP have not yet been identified. In fact, it remains unclear whether PLP is involved in the processing of ToV pp1a [21]. Moreover, the viral species of the inserted PLP sequences in porcine EV-G have not been determined, as they form a separate cluster distantly related to those of PToV, BToV, and EToV, with variable lengths observed in recombinant strains from different countries [14]. In contrast, in this study, we used the known 3CLP from PToV to identify its specific host substrate proteins, pGSDMD, and to demonstrate the resulting biological effects.

**Figure 8.**
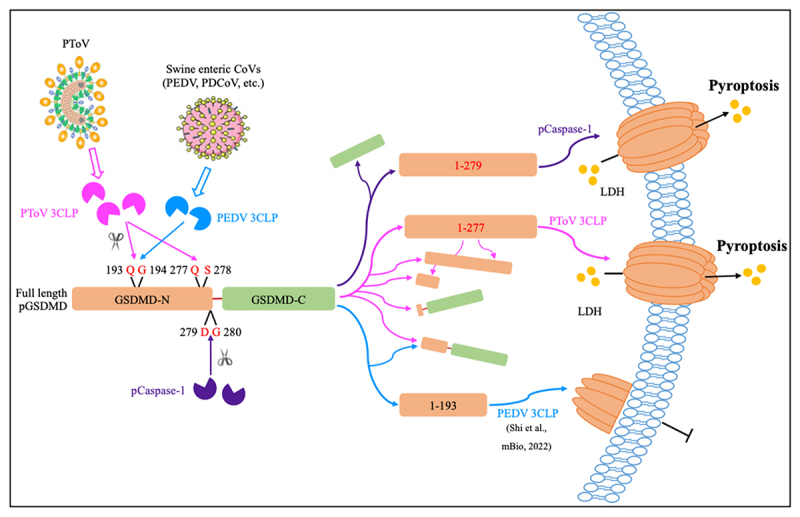
Schematic diagram illustrating the mechanism of 3CLP-mediated pGSDMD cleavage by PToV, swine enteric CoVs, or pCaspase-1, and the distinct consequences of inducing (for PToV and pCaspase-1) versus inhibiting pyroptosis (for PEDV).

Since GSDMD is the primary executor of pyroptosis by forming membrane pores with GSDMD-N (human GSDMD_1–275_ or pGSDMD_1–279_) and plays a pivotal role in the release of inflammatory cytokines (Figure 8), including bioactive IL-1β and IL-18, which contribute to antiviral immunity and the inflammatory response [38], it becomes a direct cleavage target for various viral 3C or 3CL proteases [20,31,33–35]. In most cases, as demonstrated by several human and porcine CoVs as well as human enterovirus 71, the noncanonical cleavage of GSDMD occurs at the conserved Q193 site, resulting in a nonfunctional N-terminal product that inhibits pyroptosis (Figure 8) [31,35,41]. Surprisingly, PToV 3CLP not only cleaves pGSDMD at Q193 but also at Q277; the latter cleavage appears to be dominant and mimics the behavior of pGSDMD_1–279_ (though pGSDMD_1–277_ appears to be cleaved at Q193 in further as shown in Figure 6D), resulting in the activation of pyroptosis (Figure 8). Whether the differences in protease activity and substrate recognition between CoV (cysteine protease) and ToV 3CLPs (serine protease) lead to the opposite outcomes remains to be further investigated. Intriguingly, Seneca Valley virus (SVV; a swine picornavirus) 3CP also induces pyroptosis by cleaving pGSDMD at Q193 and Q277 [32]. It is possible that SVV 3CP and PToV 3CLP share a very conserved tertiary structure to target and cleave pGSDMD, but this will need to be confirmed once the crystal structure of PToV 3CLP is resolved.

The contrast impacts of 3CLPs-mediated pGSDMD cleavage between swine enteric CoVs, exemplified by PEDV, and PToV could partly account for the divergent clinical manifestations observed in pigs infected by these nidoviruses. PEDV infection leads to severe diarrhea in newborn piglets [6], while PToV infection is considered to be either nonpathogenic or to causes only mild symptoms [14]. This divergence in clinical severity may be attributed to the differential capacities of PEDV and PToV 3CLPs to modulate pyroptosis through their specific interactions with pGSDMD. The PEDV 3CLP antagonizes pyroptosis by targeting pGSDMD at the Q193 site, a strategic cleavage that likely promotes viral proliferation and in infected cells. In contrast, PToV 3CLP triggers pyroptosis through dual cleavage at both Q193 and Q279, with the latter cleavage appearing to be dominant and form membrane pore, which may act as a natural brake on PToV dissemination within the host. Notably, the 3CLP of an equine arterivirus (EAV) also targets GSDMD to cleave a 25-kDa fragment [33]; however, the subsequent effect on cell pyroptosis has not yet been determined.

In summary, we have identified the first host cellular substrate of 3CLP from a specific ToV species. Although this substrate, pGSDMD, is a common target for CoV 3CLPs, the outcomes of its cleavage significantly differ in terms of pyroptosis. The intricate interplay between nidoviral proteases and their host cellular substrates not only regulates the cellular fate, such as pyroptotic cell death, but also shapes the potential pathogenicity and possible disease progression, offering new insights into the complex dynamics of nidovirus-host interactions [51].

## Data Availability

All data supporting the findings of this study are available in ScienceDB (http://doi.org/10.57760/sciencedb.24655).

## References

[1] Walker PJ, Siddell SG, Lefkowitz EJ, et al. Recent changes to virus taxonomy ratified by the International Committee on Taxonomy of Viruses. Arch Virol. 2022;167(11):2429–17. doi: 10.1007/s00705-022-05516-5 35999326 PMC10088433

[2] Lauber C, Zhang X, Vaas J, et al. Deep mining of the sequence read archive reveals major genetic innovations in coronaviruses and other nidoviruses of aquatic vertebrates. PLoS Pathog. 2024;20(4):e1012163. doi: 10.1371/journal.ppat.1012163 38648214 PMC11065284

[3] Cui J, Li F, Shi ZL. Origin and evolution of pathogenic coronaviruses. Nat Rev Microbiol. 2019;17(3):181–192. doi: 10.1038/s41579-018-0118-9 30531947 PMC7097006

[4] Wu A, Peng Y, Huang B, et al. Genome composition and divergence of the novel coronavirus (2019-nCoV) originating in China. Cell Host Microbe. 2020;27(3):325–328. doi: 10.1016/j.chom.2020.02.001 32035028 PMC7154514

[5] Huang YW, Meng XJ. Novel strategies and approaches to develop the next generation of vaccines against porcine reproductive and respiratory syndrome virus (PRRSV). Virus Res. 2010;154(1–2):141–149. doi: 10.1016/j.virusres.2010.07.020 20655962 PMC7132426

[6] Huang YW, Dickerman AW, Pineyro P, et al. Origin, evolution, and genotyping of emergent porcine epidemic diarrhea virus strains in the United States. MBio. 2013;4(5):e00737–13. doi: 10.1128/mBio.00737-13 24129257 PMC3812708

[7] Pan Y, Tian X, Qin P, et al. Discovery of a novel swine enteric alphacoronavirus (SeAcov) in southern China. Vet Microbiol. 2017;211:15–21. doi: 10.1016/j.vetmic.2017.09.020 29102111 PMC7117260

[8] Wang B, Liu Y, Ji CM, et al. Porcine deltacoronavirus engages the transmissible gastroenteritis virus functional receptor porcine aminopeptidase N for infectious cellular entry. J Virol. 2018;92:e0031818. 10.1128/JVI.00318-18 PMC5974500 29618640

[9] Weiss M, Steck F, Horzinek MC. Purification and partial characterization of a new enveloped RNA virus (Berne virus). J Gen Virol. 1983;64(Pt 9):1849–1858. 6886677 10.1099/0022-1317-64-9-1849

[10] Woode GN, Reed DE, Runnels PL, et al. Studies with an unclassified virus isolated from diarrheic calves. Vet Microbiol. 1982;7(3):221–240. doi: 10.1016/0378-1135(82)90036-0 7051518 PMC7117454

[11] Beards GM, Hall C, Green J, et al. An enveloped virus in stools of children and adults with gastroenteritis that resembles the Breda virus of calves. Lancet. 1984;1(8385):1050–1052. doi: 10.1016/S0140-6736(84)91454-5 6143978 PMC7173266

[12] Weiss M, Steck F, Kaderli R, et al. Antibodies to Berne virus in horses and other animals. Vet Microbiol. 1984;9(6):523–531. doi: 10.1016/0378-1135(84)90014-2 6506447 PMC7117441

[13] Dai X, Lu S, Shang G, et al. Characterization and identification of a novel torovirus associated with recombinant bovine torovirus from Tibetan antelope in Qinghai-Tibet Plateau of China. Front Microbiol. 2021;12:737753. doi: 10.3389/fmicb.2021.737753 34552576 PMC8451951

[14] Hu ZM, Yang YL, Xu LD, et al. Porcine torovirus (PToV)-a brief review of etiology, diagnostic assays and current epidemiology. Front Vet Sci. 2019;6:120. 31058174 10.3389/fvets.2019.00120 PMC6482245

[15] Durham PJ, Hassard LE, Norman GR, et al. Viruses and virus-like particles detected during examination of feces from calves and piglets with diarrhea. Can Vet J. 1989;30:876–881. 17423455 PMC1681319

[16] Pignatelli J, Jimenez M, Luque J, et al. Molecular characterization of a new PToV strain. Evol Implic Virus Res. 2009;143(1):33–43. doi: 10.1016/j.virusres.2009.02.019 PMC7114482 19463719

[17] Penrith ML, Gerdes GH. Breda virus-like particles in pigs in South Africa. J S Afr Vet Assoc. 1992;63(3):102. 1328635

[18] Pignatelli J, Jiménez M, Grau-Roma L, et al. Detection of porcine torovirus by real time RT-PCR in piglets from a Spanish farm. J Virol Methods. 2010;163(2):398–404. doi: 10.1016/j.jviromet.2009.10.031 19887084

[19] Shin DJ, Park SI, Jeong YJ, et al. Detection and molecular characterization of porcine toroviruses in Korea. Arch Virol. 2010;155(3):417–422. doi: 10.1007/s00705-010-0595-2 20127374 PMC7087203

[20] Fujii Y, Kashima Y, Sunaga F, et al. Complete genome sequencing and genetic analysis of a Japanese porcine torovirus strain detected in swine feces. Arch Virol. 2020;165(2):471–477. doi: 10.1007/s00705-019-04514-4 31863265

[21] Ujike M, Taguchi F. Recent progress in torovirus molecular biology. Viruses. 2021;13(3):435. doi: 10.3390/v13030435 33800523 PMC7998386

[22] Ziebuhr J, Snijder EJ, Gorbalenya AE. Virus-encoded proteinases and proteolytic processing in the nidovirales. J Gen Virol. 2000;81(4):853–879. doi: 10.1099/0022-1317-81-4-853 10725411

[23] Fang Y, Snijder EJ. The PRRSV replicase: exploring the multifunctionality of an intriguing set of nonstructural proteins. Virus Res. 2010;154:61–76. 20696193 10.1016/j.virusres.2010.07.030 PMC7114499

[24] Smits SL, Snijder EJ, de Groot RJ. Characterization of a torovirus main proteinase. J Virol. 2006;80(8):4157–4167. doi: 10.1128/JVI.80.8.4157-4167.2006 16571831 PMC1440467

[25] Snijder EJ, Wassenaar AL, van Dinten LC, et al. The arterivirus nsp4 protease is the prototype of a novel group of chymotrypsin-like enzymes, the 3C-like serine proteases. J Biol Chem. 1996;271(9):4864–4871. doi: 10.1074/jbc.271.9.4864 8617757

[26] Anand K. Structure of coronavirus main proteinase reveals combination of a chymotrypsin fold with an extra alpha-helical domain. Embo J. 2002;21(13):3213–3224. doi: 10.1093/emboj/cdf327 12093723 PMC126080

[27] Hegyi A, Ziebuhr J. Conservation of substrate specificities among coronavirus main proteases. J Gen Virol. 2002;83(3):595–599. doi: 10.1099/0022-1317-83-3-595 11842254

[28] Xu S, Zhou J, Chen Y, et al. Characterization of self-processing activities and substrate specificities of porcine torovirus 3C-like protease. J Virol. 2020;94(20):e0128220. doi: 10.1128/JVI.01282-20 PMC7527039 32727876

[29] Ng CS, Stobart CC, Luo H. Innate immune evasion mediated by picornaviral 3C protease: possible lessons for coronaviral 3C-like protease? Rev Med Virol. 2021;31(5):1–22. doi: 10.1002/rmv.2206 PMC7883238 33624382

[30] Sun D, Chen S, Cheng A, et al. Roles of the picornaviral 3c proteinase in the viral life cycle and host cells. Viruses. 2016;8(3):82. doi: 10.3390/v8030082 26999188 PMC4810272

[31] Shi F, Lv Q, Wang T, et al. Coronaviruses nsp5 antagonizes porcine gasdermin D-mediated pyroptosis by cleaving pore-forming p30 fragment. mBio MBio. 2022;13(1):e0273921. doi: 10.1128/mbio.02739-21 35012343 PMC8749417

[32] Wen W, Li X, Wang H, et al. Seneca Valley virus 3C protease induces pyroptosis by directly cleaving porcine gasdermin D. J Immunol. 2021;207(1):189–199. doi: 10.4049/jimmunol.2001030 34183365

[33] Zhao G, Li T, Liu X, et al. African swine fever virus cysteine protease pS273R inhibits pyroptosis by noncanonically cleaving gasdermin D. J Biol Chem. 2022;298(1):101480. doi: 10.1016/j.jbc.2021.101480 34890644 PMC8728581

[34] Yamaoka Y, Matsunaga S, Jeremiah SS, et al. Zika virus protease induces caspase-independent pyroptotic cell death by directly cleaving gasdermin D. Biochem Biophys Res Commun. 2021;534:666–671. doi: 10.1016/j.bbrc.2020.11.023 33208231

[35] Planes R, Pinilla M, Santoni K, et al. Human NLRP1 is a sensor of pathogenic coronavirus 3CL proteases in lung epithelial cells. Mol Cell. 2022;82(13):2385–400 e9. doi: 10.1016/j.molcel.2022.04.033 35594856 PMC9108100

[36] Shi J, Gao W, Shao F. Pyroptosis: gasdermin-mediated programmed necrotic cell death. Trends Biochem Sci. 2017;42(4):245–254. doi: 10.1016/j.tibs.2016.10.004 27932073

[37] Bergsbaken T, Fink SL, Cookson BT. Pyroptosis: host cell death and inflammation. Nat Rev Microbiol. 2009;7(2):99–109. doi: 10.1038/nrmicro2070 19148178 PMC2910423

[38] Sborgi L, Rühl S, Mulvihill E, et al. GSDMD membrane pore formation constitutes the mechanism of pyroptotic cell death. Embo J. 2016;35:1766–1778. 27418190 10.15252/embj.201694696 PMC5010048

[39] Kayagaki N, Stowe IB, Lee BL, et al. Caspase-11 cleaves gasdermin D for non-canonical inflammasome signalling. Nature. 2015;526(7575):666–671. doi: 10.1038/nature15541 26375259

[40] Man SM, Karki R, Kanneganti TD. Molecular mechanisms and functions of pyroptosis, inflammatory caspases and inflammasomes in infectious diseases. Immunol Rev. 2017;277(1):61–75. doi: 10.1111/imr.12534 28462526 PMC5416822

[41] Lei X, Zhang Z, Xiao X, et al. Enterovirus 71 inhibits pyroptosis through cleavage of gasdermin D. J Virol. 2017;91(18):e0106917. doi: 10.1128/JVI.01069-17 PMC5571240 28679757

[42] Qin P, Yang YL, Hu ZM, et al. A novel spike subunit 1-based enzyme-linked immunosorbent assay reveals widespread porcine torovirus infection in eastern China. Transbound Emerg Dis. 2022;69(2):598–608. doi: 10.1111/tbed.14026 33555108

[43] Yan Q, Wu L, Chen L, et al. Vesicular stomatitis virus-based vaccines expressing EV71 virus-like particles elicit strong immune responses and protect newborn mice from lethal challenges. Vaccine. 2016;34(35):4196–4204. doi: 10.1016/j.vaccine.2016.06.058 27373596

[44] Jiao Y, Zhao P, Xu LD, et al. Enteric coronavirus nsp2 is a virulence determinant that recruits NBR1 for autophagic targeting of TBK1 to diminish the innate immune response. Autophagy. 2024;20:1762–1779. 38597182 10.1080/15548627.2024.2340420 PMC11262224

[45] Hu JJ, Liu X, Xia S, et al. Fda-approved disulfiram inhibits pyroptosis by blocking gasdermin D pore formation. Nat Immunol. 2020;21(7):736–745. doi: 10.1038/s41590-020-0669-6 32367036 PMC7316630

[46] Wang S, Dai T, Qin Z, et al. Targeting liquid-liquid phase separation of SARS-CoV-2 nucleocapsid protein promotes innate antiviral immunity by elevating MAVS activity. Nat Cell Biol. 2021;23(7):718–732. doi: 10.1038/s41556-021-00710-0 34239064

[47] Lin J, Liu F, Gao F, et al. Vesicular stomatitis virus sensitizes immunologically cold tumors to checkpoint blockade by inducing pyroptosis. Biochim Biophys Acta Mol Basis Dis. 2022;1868(12):166538. doi: 10.1016/j.bbadis.2022.166538 36096276

[48] Liu X, Zhang Z, Ruan J, et al. Inflammasome-activated gasdermin D causes pyroptosis by forming membrane pores. Nature. 2016;535(7610):153–158. doi: 10.1038/nature18629 27383986 PMC5539988

[49] Draker R, Roper RL, Petric M, et al. The complete sequence of the bovine torovirus genome. Virus Res. 2006;115(1):56–68. doi: 10.1016/j.virusres.2005.07.005 16137782 PMC7114287

[50] Shang P, Misra S, Hause B, et al. A naturally occurring recombinant enterovirus expresses a torovirus deubiquitinase. J Virol. 2017;91(14):e0045017. doi: 10.1128/JVI.00450-17 PMC5487566 28490584

[51] Pan D. Cleavage of cellular substrate porcine gasdermin D by porcine torovirus 3C-like protease induces pyroptosis. 2025. doi: 10.57760/sciencedb.24655 PMC12758170 41400836

